# Glycemic variability through the perspective of the glycemia risk index and time in range and their association with glycated hemoglobin A1c in pediatric patients on sensor-augmented pump therapy

**DOI:** 10.3389/fendo.2024.1388245

**Published:** 2024-06-18

**Authors:** Gordana Bukara-Radujkovic, Vesna Miljkovic

**Affiliations:** ^1^ Pediatric Clinic, University Clinical Center of the Republic of Srpska, Banja Luka, Bosnia and Herzegovina; ^2^ Pediatric Department, Faculty of Medicine, University of Banja Luka, Banja Luka, Bosnia and Herzegovina

**Keywords:** glycemia risk index, glycemic variability, continuous glucose monitoring, pediatric diabetes, time in range

## Abstract

**Introduction:**

From the introduction of continuous glucose monitoring (CGM) in treatments of type 1 diabetes, particularly its integration with insulin pumps, there has been a quest for new parameters that describe optimal glycemic control. As of the consensus reached in 2019, the ambulatory glucose profile (AGP) has become the standard, with time in range (TIR) emerging as a fundamental parameter for metabolic control assessment. However, with technological advancements, new parameters, such as the glycemia risk index (GRI), have been introduced and clinically utilized. Therefore, exploring the relationships between traditional and novel parameters to understand metabolic control comprehensively is imperative.

**Materials and methods:**

This study was conducted at the Pediatric Clinic of the University Hospital of the Republic of Srpska Banja Luka between January and July 2023. The participants were randomly selected, with the inclusion criteria specifying an age greater than eight years and a diabetes type 1 duration exceeding two years. All participants were required to use a sensor-augmented insulin pump for the next three months (90 days), irrespective of prior use, with the suspend-before-low option activated.

**Results:**

Of the 35 participants, 30 completed the study, 14 (46.7%) of whom were male. The mean age of the subjects was 14.90 ± 2.88 years, and the mean duration of diabetes was 7.83 ± 4.76 years. Over the 90-day period, HbA1c increased to an average of 7.31%. The analysis revealed significant effects of TIR (β=-0.771) and GRI (β=0.651) on HbA1c. Furthermore, GRI and TIR strongly correlated (β=-0.953).

**Discussion and conclusion:**

New parameters generated from the ambulatory glucose profile (AGP) can help clinicians create a complete picture of a patient’s metabolic control in relation to HbA1c levels. Additionally, the GRI is a mathematically tailored parameter that incorporates all components of the ambulatory glucose profile and demonstrates strong correlations with laboratory-measured HbA1c and TIR. The GRI potentially can become a valuable statistical parameter for evaluating and managing patients in routine clinical practice.

## 1 Introduction

Since 2019, after the recommendations of the international consensus on time in range (1) were announced and utilized, the management of diabetes in children and adolescents has encountered significant challenges. Although we are constantly discussing the increased penetration of continuous glucose monitoring technology (CGM), particularly among pediatric populations, glycated hemoglobin A1c (HbA1c) remains the primary parameter for assessing metabolic control in many resource-limited settings and among patients who decline or struggle with CGM technology (2). Global data indicate that only 37% of individuals with type 1 diabetes achieve an HbA1c below 7.50%, with a mere 21% reaching or maintaining levels at or below 7.00%, as recommended by consensus guidelines (3).

The utilization of CGM can mitigate the limitations associated with HbA1c, which include variability in laboratory measurements influenced by various pathological (e.g., anemia, uremia, hemoglobinopathies) and physiological (e.g., pregnancy) factors, as well as the inability to capture daily glycemic fluctuations (3). CGM, particularly when focusing on the time in range (TIR) parameter (i.e., time spent within the target range of 3.9–10 mmol/L or 70–180 mg/dL), is becoming the standard of use in clinical practice, due to its ability to bypass the shortcomings of HbA1c and effectively depict daily glucose fluctuations, thereby reducing glycemic variability—a significant contributor to oxidative stress, particularly in children (4). Recently, TIR has been endorsed as a key parameter for clinical trials (5, 6). However, in addition to TIR, other parameters derived from ambulatory glucose profile (AGP), such as time below range (TBR), time above range (TAR), and coefficient of variation (CV), provide clinicians with valuable insights into glycemic variability beyond the confines of TIR and HbA1c (7, 8).

Because of the multitude of parameters derived from AGP essential for comprehensive glycemic assessment, researchers have recently proposed that the parameters of time above and below the target range should be integrated into a new parameter, the glycemia risk index (GRI). This parameter was generated mathematically from the TBR and TAR values, as already stated in the ambulatory glucose profile. It contains a hypoglycemic component (CHypo) that is twice as significant as the hyperglycemic component (CHyper). Theoretically, lower GRI values, ranging from 0 to 100, correlate with reduced risk of hypoglycemia and hyperglycemia. Contrastingly, the lower the GRI, the higher the TIR (9).

The well-established correlation between HbA1c and TIR (10, 11) prompts further investigation of the relationship between these parameters and GRI across different therapeutic modalities. Therefore, our goal with this study is to explore the interdependence of HbA1c levels and variability parameters derived from ambulatory glucose profile including GRI components, in a pediatric population undergoing sensor-augmented pump therapy.

## 2 Materials and methods

**Figure 1:**
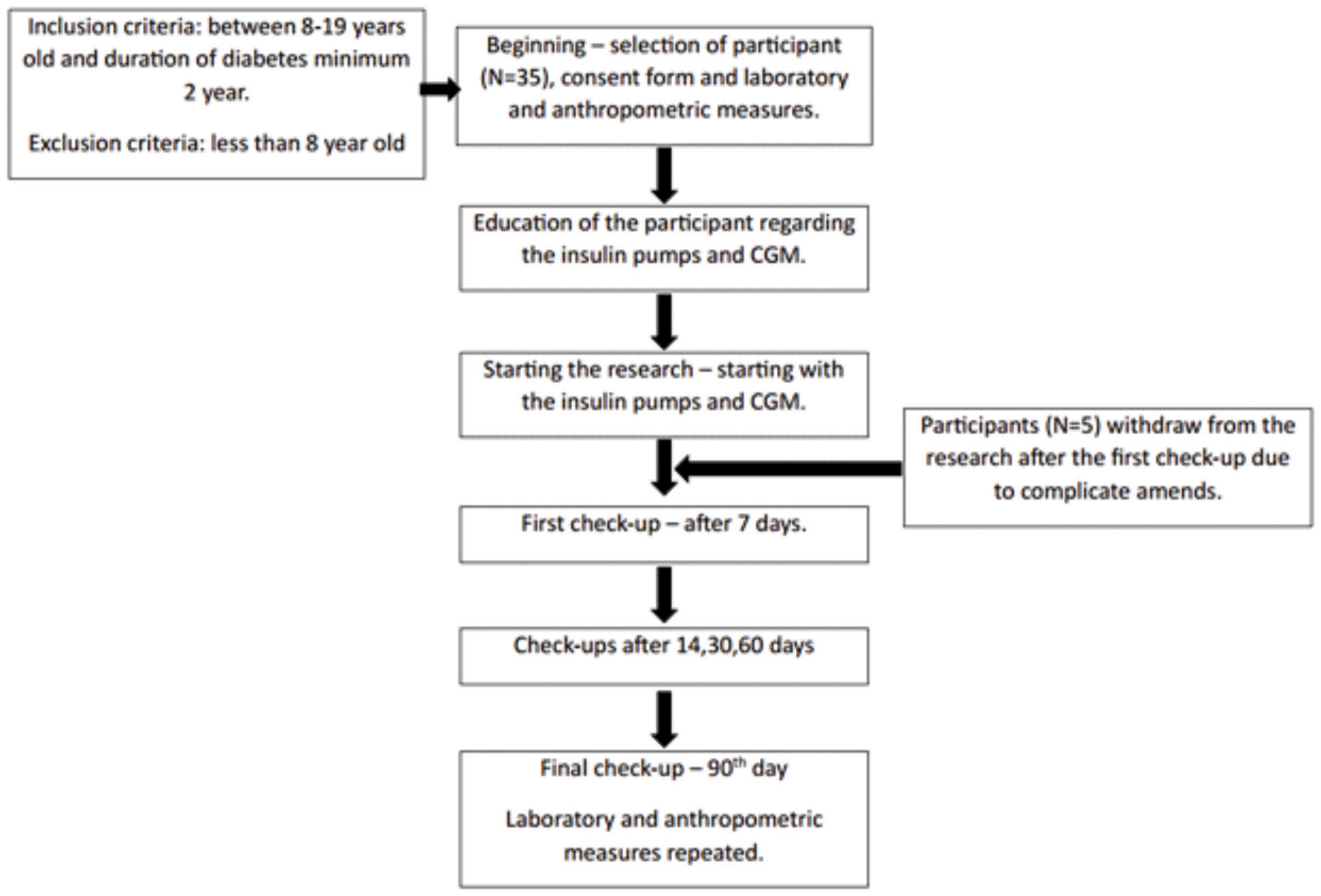
Research scheme.

This study was conducted at the Pediatric Clinic of the University Hospital of the Republic of Srpska, Banja Luka, between January and July 2023. Participants were randomly selected, with every fifth eligible patient attending regular clinic visits being invited to participate. The inclusion criteria were age > 8 years and diabetes type 1 duration exceeding two years. Exclusion criteria included diabetes type 1 duration less than two years. Informed consent was obtained from all participants or their legal guardians. Participants were required to utilize a sensor-augmented insulin pump for the next 3 months from the baseline, no matter of prior use of insulin pump or the CGM, with the suspend before low feature enabled, and attend regular check-ups. The low threshold set was 4.0 mmol/L for all participants, as it is presented in **Figure 1**. Diagnostic analyses were conducted in accordance with the Declaration of Helsinki, with HbA1c and anthropometric measurements recorded at baseline and after 3 months.

### 2.1 Statistical analysis

Numerical variables were described using measures of central tendency, mean value, standard deviation, or minimum and maximum values. Categorical variables are described as frequencies (%) of the total sample. The IBM SPSS 22 statistical program was used for statistical analysis. Pearson’s correlation coefficient was used to measure the dependence between the numerical variables. The dependence of the numerical variables on time was determined using a t-test for paired samples. A linear regression model was set where the dependent variable was HbA1c at the end of the study, and the independent variables were parameters from AGP or derived from it such as GRI. Statistical significance was set at p < 0.05. GRI was calculated using the mathematical formula, and the component of GRI was also considered a significant variable in the statistical analysis, replacing the TBR, levels 1 and 2, and TAR, levels 1 and 2 (9). Other AGP parameters were used in the analysis, and recommendations from the consensus regarding parameter targets in clinical care were used (1).

## 3 Results

Thirty of the 35 initially enrolled participants successfully completed the study, with 46.7% male participants. The mean age was 14.90 ± 2.88 years, and the mean duration of diabetes was 7.83 ± 4.76 years. Within this cohort, only two participants (6.7%) were diagnosed with celiac disease in addition to type 1 diabetes mellitus. Laboratory analyses and anthropometric measurements were conducted at baseline and after a 90-day period.

No statistically significant changes were observed in BMI (from 20.80 ± 3.76 BMI to 20.94 ± 3.30) after three months of utilizing sensor-augmented pump therapy. However, there was an increase in the average value of laboratory-measured HbA1c levels compared with the baseline measurement. HbA1c levels increased from 7.17 ± 0.92% to 7.31 ± 0.74%. These two parameters exhibited a positive correlation, with a Pearson’s correlation coefficient of 0.542 (p<0.05). The paired-sample t-test for this variable did not yield statistically significant results (t=-0.945, p=0.352).

Further analysis involved dividing the subjects into two groups based on their initial HbA1c values: those with HbA1c values equal to or less than 7.00% and those with initial HbA1c values higher than 7.00%. Fourteen participants had HbA1c values less than or equal to 7.00%, and a t-test comparison within this group demonstrated a statistical significance (correlation = 0.847, p=0.0001; paired sample t-test: t=-5.090, p=0.0002). In this group, HbA1c increased by an average of 0.59 ± 0.44%. Conversely, no statistically significant reduction was observed in the group where HbA1c was over 7.00%, despite an initial average HbA1c of 7.78 ± 0.76% decreasing to 7.53 ± 0.68%. The correlation between the variables within this group was not significant (correlation = 0.274, p=0.305; t-test: t=1.184, p=0.255). Therefore, the overall correlation between HbA1c levels at the beginning and end of the study was primarily influenced by a statistically significant increase in the group with initial HbA1c values of less than 7.00%.

The variables obtained from the ambulatory glucose profile (AGP) are presented in **Table 1**, including the hypoglycemic and hyperglycemic components necessary for calculating the Glycemia Risk Index (GRI). Established formula was used to calculate the GRI, and its components (9). The results are provided by month to observe changes, as well as an average for the entire 90-day period.

**Table 1:** Values of variables obtained from ambulatory glucose profile and GRI.

_Variable_	_1_^st^_month_	_2_^nd^_month_	_3_^rd^_month_	_Average for 90 days_
** _TIR_ **	68.43 ± 10.70	69.13 ± 10.88	71.40 ± 8.59	69.66 ± 8.96
** _CHypo_ **	2.05 ± 1.74	1.95 ± 1.39	2.20 ± 1.71	2.07 ± 1.44
** _CHyper_ **	17.75 ± 7.06	17.45 ± 7.64	15.62 ± 5.88	16.94 ± 6.10
** _GRI_ **	34.56 ± 13.05	33.78 ± 11.91	31.59 ± 11.15	33.31 ± 10.71
** _CV_ **	34.28 ± 4.63	34.00 ± 3.13	34.02 ± 4.11	34.10 ± 3.65
** _GMI_ **	7.04 ± 0.32	7.02 ± 0.37	6.94 ± 0.28	7.00 ± 0.28
** _MEAN SG_ **	8.66 ± 0.79	8.60 ± 0.86	8.43 ± 0.66	8.56 ± 0.68
** _TDD_ **	44.45 ± 19.18	45.27 ± 19.76	45.77 ± 18.97	45.16 ± 19.21

Values are presented as mean ± standard deviation. TIR, Time In Range (from ambulatory glucose profile); CHypo, Hypoglycemic component of GRI (mathematically calculated) (9); CHyper, Hyperglycemic component of GRI (mathematically calculated) (9); GRI, Glycemia Risk Index (mathematically calculated) (9); CV, Coefficient of Variation (from ambulatory glucose profile); GMI, Glucose Management Indicator (from ambulatory glucose profile); SG, Sensor Glucose Value; TDD, Total Daily Dose of insulin.

Analysis of these parameters revealed that they approached the clinical targets by the third month, as set by consensus. The correlations between these parameters and laboratory-measured HbA1c levels at the end of the research period were examined and are presented in **Table 2**. Most parameters obtained from the AGP and GRI, along with their components, demonstrated high correlations (>0.65 and p<0.001), except for the hypoglycemic component of the GRI, coefficient of variation (CV), and total daily dose (TDD), which had p values greater than 0.05.

**Table 2:** Pearson correlation between HbA1c and parameters derived from AGP.

Variable	HbA1c after 90 days	
	Pearson correlation coefficient	p-value
**TIR**	-0.771	p<0.001
**Chypo**	-0.124	p=0.515
**Chyper**	0.770	p<0.001
**GRI**	0.651	p<0.001
**CV**	0.113	p=0.552
**GMI**	0.810	p<0.001
**Mean SG**	0.782	p<0.001
**TDD**	0.040	p=0.835

AMP, Ambulatory Glucose profile1; HbA1c, Glycated hemoglobin A1c; TIR, Time In Range (from ambulatory glucose profile); CHypo, Hypoglycemic component of GRI (mathematically calculated) (9); CHyper, Hyperglycemic component of GRI (mathematically calculated) (9); GRI, Glycemia Risk Index (mathematically calculated) (9); CV, Coefficient of Variation (from ambulatory glucose profile); GMI, Glucose Management Indicator (from ambulatory glucose profile); SG, Sensor Glucose Value; TDD, Total Daily Dose of insulin.

Average glycemia and GMI, although highly correlated with HbA1c, were not considered predictor variables in the linear regression analysis. Our analysis focused on the relationship between the GRI and its components and TIR and HbA1c levels. The results of this linear regression analysis were R=0.822, R^2^ = 0.676, and adjusted R^2^ = 0.639, indicating that 63.9% of the HbA1c distribution could be explained by these variables (F=18.092, p<0.001). The coefficients of the TIR (t=-2.290, p<0.05) and GRI (t=-2.468, p<0.05) were statistically significant.

**Figure 2:**
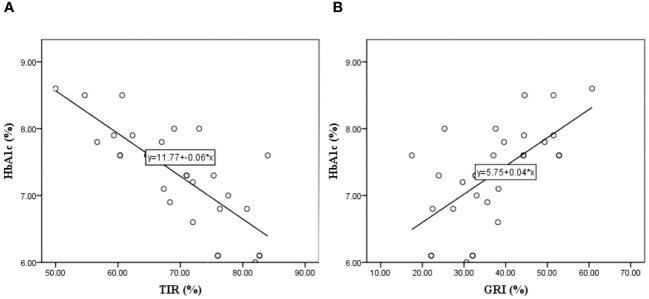
Linear regression model where the HbA1c is dependent variable and the Time In Range – TIR **(A)**, and Glycemia Risk Index – GRI **(B)** are independent variable.

In further analysis, we wanted to examine the individual influence of the GRI and TIR on HbA1c; therefore, we performed a single linear regression of these variables with HbA1c as dependent variable. In this regression, the GRI was statistically significant R=0.651, R^2^ = 0.423, and adjusted R^2^ = 0.403 (F=20.563, t=16.671, p<0.001). Standardized coefficient β=0.651, i.e., if GRI increases by 1 standard deviation, HbA1c increases by 0.651 standard deviations. Also, TIR shows statistical significance R=0.771, R^2^ = 0.594, and adjusted R^2^ = 0.580 (F=41.028, t=16.677, p<0.001) and standardized coefficient β=-0.771, which means if TIR increases by 1 standard deviation, HbA1c decreases by 0.771 standard deviation. The **Figure 2** shows that with an increase in HbA1c, the TIR decreased, and the GRI increased.

**Figure 3:**
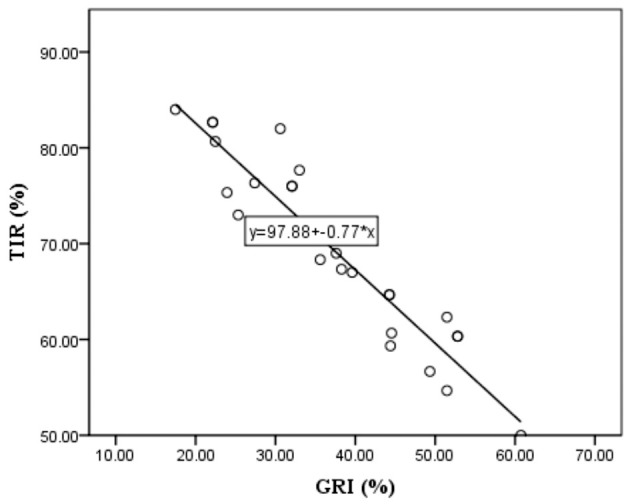
Linear regression model of Time In Range - TIR as dependent and Glycemia Risk Index - GRI as independent variable.

We examined the relationship between the parameters obtained from the AGP and the GRI. The correlations of the GRI components with the variability parameters from AGP is presented in **Table 3**. There is a negative correlation between GRI, CHyper, and TIR, which is logical because a higher TIR indicates a lower GRI and less hyperglycemic component of the GRI. There was no correlation between the TIR and hypoglycemic components. Multivariate linear regression was performed with the GRI as the dependent variable to examine which variability parameters had the greatest influence on the GRI. The model showed a high statistical significance, R=0.991, R^2^ = 0.983, and adjusted R^2^ = 0.979, F=271.162, p<0.001. This means that 98.3% of the GRI distribution can be explained by TIR, CV, GMI, average SG, and TDD. The coefficients for the variables TIR, CV, and TDD show statistical significance in the model, so their standardized values and t-test values are as follows: β=-0.969, t=-8.162, p<0.001 for TIR, β=0.193, t=4.178, p< 0.001 for CV and β=0.107, t=2.986, p<0.006 for TDD. Because the values for TIR were the highest, a single linear regression analysis of the dependence of the GRI on TIR was performed. The obtained results of R=0.953, R^2^ = 0.909, and adjusted R^2^ = 0.906, F=280.002, t=-16.733, and standardized β=-0.953 at the significance level p<0.001 tell us that an increase in TIR by 1 standard deviation leads to a decrease in GRI for 0.953 standard deviation in our cohort. It is observed that with the increase in GRI comes the reduction of TIR and vice versa, as it can been seen in **Figure 3**. In our examined group, the highest GRI is close to 60, and the respondent TIR is approximately 50.

**Table 3:** Correlation of variability parameters from AGP and GRI and its components.

Variable	GRI		Chyper		Chypo	
	Pearson correlation coefficient	p-value	Pearson correlation coefficient	p-value	Pearson correlation coefficient	p-value
**TIR**	-0.953	p<0.001	-0.977	p<0.001	-0.158	p=0.405
**CV**	0.687	p<0.001	0.446	p<0.05	0.686	p<0.001
**GMI**	0.774	p<0.001	0.949	p<0.001	-0.224	p=0.233
**Mean SG**	0.800	p<0.001	0.961	p<0.001	-0.186	p=0.325
**TDD**	0.404	P<0.05	0.183	P=0.334	-0.589	p<0.001

AMP, Ambulatory Glucose profile1; GRI , Glycemia Risk Index (mathematically calculated) (9); CHypo , Hypoglycemic component of GRI (mathematically calculated) (9); CHyper , Hyperglycemic component of GRI (mathematically calculated) (9); TIR , Time In Range (from ambulatory glucose profile); CV , Coefficient of Variation (from ambulatory glucose profile); GMI , Glucose Management Indicator (from ambulatory glucose profile); SG , Sensor Glucose Value; TDD , Total Daily Dose of insulin.

## 4 Discussion

The sensor-augmented pump, which uses an algorithm that predicts and automatically suspends insulin delivery before reaching a low threshold, set in our study on 4.0 mmol/L, has shown significant benefits over its period of use (12). In our study group, the utilization of this system led to an increase in the parameters derived from the ambulatory glucose profile, culminating in the achievement of consensus-recommended values by the third month (1). Interestingly, in the third month, in addition to the increase in TIR and decrease in CHyper, there was also an increase in CHypo. This can be explained by the fact that the participants began to trust the system and allowed the prediction algorithm to execute fully (13), not correcting the hypoglycemia with carbs on values higher than 4.0 mmol/L. The sensor augmented pump system, designed to suspend insulin delivery 30 minutes before reaching a low threshold of 4.0 mmol/L, allowed users to intervene promptly and prevent hypoglycemic events. As participants familiarized themselves with the system’s functionality, there was a noticeable decrease in hypoglycemic incidents during the initial month, followed by a subsequent increase by the third month as users gained confidence in the system’s reliability. Over time, the diminishing fear of hypoglycemia, a pivotal psychological component in diabetes management (14), leads to a decreased tendency to react promptly to and correct hypoglycemic episodes upon activation of the algorithm. This behavior typically results in reactive hyperglycemia prior to the adoption of this system (15). Notably, this system effectively mitigated hypoglycemia, as evidenced by the absence of statistical significance in variables describing hypoglycemia (CHypo) in the ambulatory glucose profile and Glycemia Risk Index (GRI) and their lack of association with other examined variables or HbA1c.

Although the HbA1c levels showed a nominal increase across the entire group, this increase was not statistically significant. However, participants with initial HbA1c levels of < 7.00% at baseline displayed a statistically significant increase, whereas the other groups exhibited a non-significant decrease. The observed elevation in HbA1c among participants with initially lower HbA1c levels can be attributed to the aforementioned system and the reduction of hypoglycemic components, suggesting that the initially lower HbA1c levels in this group may have been due to the prior hypoglycemia (16). Given the absence of prior continuous glucose monitoring data, the direct causation of the lower baseline HbA1c cannot be definitively established. However, increasing the HbA1c, especially in the group where baseline values were lower than 7.0, suggested a potential association.

HbA1c demonstrated the highest correlation with the average mean sensor glucose value (17), consistent with previous studies. Surprisingly, the coefficient of variation (CV) did not significantly correlate with HbA1c (18), indicating that this parameter, which characterizes daily glycemic variability, has no notable effect on HbA1c, a long-term prognostic indicator of glycemic control. Our analysis primarily aimed to ascertain whether TIR and GRI, incorporating a hyperglycemic component, could adequately substitute for HbA1c in the long-term prognosis of glycemic control (19) and to elucidate the relationship between these parameters in our cohort. Our findings indicate that a 0.65 standard deviation increase in the GRI corresponds to a one standard deviation increase in HbA1c. This direct proportional relationship is dictated by the hyperglycemic component of the GRI, with the hypoglycemic component exerting no influence. Since the hypoglycemic component has no influence, we can say that the dependence on GRI and HbA1c dictates the hyperglycemic component, and it is logical that an increase in GRI leads to an increase in HbA1c.

In contrast to the GRI, the TIR is inversely proportional to HbA1c levels (20). A decrease in TIR by 0.771 standard deviations corresponded to a 1.00 standard deviation increase in HbA1c. Considering the recommendation that a TIR of 70% approximately corresponds to an HbA1c of 7.00% (10), in our study, where the TIR after 90 days was 69.66% and the HbA1c was 7.31%, a 0.36% decrease in TIR would theoretically result in a 0.47% increase in HbA1c levels. However, this discrepancy does not align with laboratory measurements of HbA1c, underscoring the inadequacy of TIR alone as a variable for forecasting long-term consequences, similar to HbA1c (21). Consequently, to evaluate metabolic control comprehensively, all variables, including TIR and GRI, must be considered, prompting us to investigate the extent of their mutual dependence in our cohort.

Our results revealed a strong correlation: a decrease in TIR by 0.953 standard deviations corresponded to a 1.00 standard deviation increase in GRI. Intriguingly, the GRI exhibited correlations with all parameters from the ambulatory glucose profile (22), as well as with CV. CV, traditionally regarded as a parameter describing glucose variability (23), did not exhibit a statistical significance with the laboratory-measured parameters in our study. Introducing the GRI as a new variability parameter is promising for overcoming this limitation (24). Furthermore, in addition to CV and TIR, the GRI demonstrated a dependence on the total daily insulin dose in our study, indicating its correlation with and dependence on the values derived from all parameters of the ambulatory glucose profile.

As the latest parameter derived from ambulatory glucose profiles, the Glycemia Risk Index (GRI) has yet to find widespread use in daily clinical practice. Studies such as this one, which integrate traditional metrics like HbA1c with modern indicators such as TIR, CV, and other AGP-derived parameters, along with potential future additions like the GRI, play a vital role in advocating for the incorporation of GRI into routine clinical assessments. This article’s significance lies in its contribution towards bridging the gap between research findings and practical implementation, thereby enhancing the clinical utility of GRI. Although limited by its small sample size, our study yielded statistically significant results. The primary limitation of our study was the financial constraints, which restricted the number of participants. Nevertheless, the statistical dependencies obtained serve as a solid foundation for future research on potentially larger scales, increasing the sample size, which can yield the statistically stronger conclusions.

## 5 Conclusion

The GRI is a mathematically tailored variable that, in our research, demonstrated statistical correlations with all AGP parameters and was highly correlated with laboratory-measured HbA1c. The GRI could potentially become a valuable statistical parameter for assessing and managing patients in routine clinical practice. Glycemic variability is a complex phenomenon with significant implications for metabolic control in the short and long term (25). This concept often remains unclear to patients, and since 2019, the dominant parameter describing it, the CV, has caused more confusion than clarity. Therefore, introducing new variables, such as the GRI, that can simplify glycemic variability for clinicians and patients may help alleviate this issue and enhance metabolic control, which is the primary objective. A comprehensive review and evaluation of all parameters, including TIR, along with other elements of AGP and GRI, can provide an accurate and complete understanding of the patient’s metabolic control.

## Data availability statement

The raw data supporting the conclusions of this article will be made available by the authors, without undue reservation.

## Ethics statement

The studies involving humans were approved by Ethics Committee of University Clinical Center of the Republic of Srpska (protocol number: 01-19-82-2/23 from March 22, 2023). The studies were conducted in accordance with the local legislation and institutional requirements. Written informed consent for participation in this study was provided by the participants’ legal guardians/next of kin.

## Author contributions

GB-R: Conceptualization, Data curation, Formal analysis, Funding acquisition, Investigation, Methodology, Project administration, Resources, Software, Supervision, Validation, Visualization, Writing – original draft, Writing – review & editing. VM: Investigation, Project administration, Writing – review & editing.
